# The effects of an integrated training program on jump performance and knee valgus in youth football players: a randomised controlled group study

**DOI:** 10.1186/s13102-025-01266-4

**Published:** 2025-07-28

**Authors:** Mehmet Behzat Turan, Aydın Pekel, Vesile Sahiner Guler, Mehmet Akif Kurt, Osman Pepe, Yahya Polat

**Affiliations:** 1 Department of Recreation, Faculty of Sports Sciences, Erciyes University, Kayseri, Turkey; 2 Department of Sports Management, Faculty of Sports Sciences, Marmara University, İstanbul, Turkey; 3 Department of Physical Education and Sports, Health Sciences Institute, Erciyes University, Kayseri, Turkey; 4 Department of Sports Management, Faculty of Sports Sciences, Suleyman Demirel University, Isparta, Turkey; 5 Department of Physical Education and Sports, Faculty of Sports Sciences, Erciyes University, Kayseri, Turkey

**Keywords:** Football, Jump performance, Anterior cruciate ligament, Knee valgus, Injury prevention, Exercise training program

## Abstract

**Background:**

This study aimed to examine the effects of stretching, strength, and plyometric exercise programs, implemented in addition to regular training, on jumping performance and knee valgus angles in football players.

**Methods:**

Participants were randomly assigned to an experimental or a control group. The experimental group engaged in an eight-week program that included stretching, strength, and plyometric exercises, while the control group continued their standard training regimen. Jumping performance variables, including Countermovement Jump (CMJ), Free CMJ, Drop Jump (DJ), Squat Jump (SJ), Horizontal Jump, and right and left knee valgus angles, were assessed at three different time points. Demographic characteristics were measured using a scale and meter, and the “My Jump 2” application was used for jump tests and knee valgus evaluations. Data were analyzed using SPSS 27. Repeated Measures ANOVA with Bonferroni post hoc comparisons was employed to analyze within-group changes, while Independent Samples T-tests were used to assess between-group differences.

**Results:**

Both groups demonstrated increased jumping performance following the intervention; however, the experimental group showed significantly greater increases across all jump parameters. Furthermore, while reductions in right and left knee valgus angles were observed in both groups, these reductions were more pronounced in the experimental group.

**Conclusion:**

The findings indicate that supplementing regular training with stretching, strength, and plyometric exercises significantly enhances jumping performance and more effectively reduces knee valgus angles in football players. These results suggest that such complementary training programs can play a critical role in improving athletic performance and reducing injury risk.

**Trial registration:**

The randomized controlled trial was registered on 06 June 2025 at ClinicalTrials.gov, under the registration number NCT07009041.

## Introduction

Since the importance of the knee joint in football players is fundamental and football is a contact sport that requires agility, jumping, running, quick changes of direction and kicking, all these activities put significant stress in the knee joint and combined with the intensity of the game, can lead to both serious and minor injuries that can affect the player’s career. Risk factors for knee injuries can be divided into non-modifiable and modifiable factors. The modifiable factors include muscle development, fitness, flexibility, balance, strength, and motor coordination [1]. Since football uses the feet and places a high load on the lower extremities, most injuries observed in football players occur in the lower extremities and knee joints [2]. It has been reported that most knee joint injuries involve the anterior cruciate ligament [3]. Causes of ACL injuries are believed to result from the complex interaction of multiple risk factors, including environmental, anatomical, hormonal, neuromuscular, and familial factors. Dynamic factors that affect the tension in the anterior cruciate ligament include knee kinematics (such as knee flexion, alignment, and movements in the frontal and transverse planes) and the knee rotational moment. ACL injuries are thought to result from unsuccessful postural adjustments and abnormal loading around the knee in response to rapid changes in the external environment [4]. Adequate prevention will improve many players’ quality of life and reduce the healthcare system’s financial burden. Developing an appropriate prevention program requires identifying injury risk factors, understanding the mechanisms of the ACL and other knee joint structures, and adapting to lifestyle, training, and time constraints among professional players, whose daily challenges are challenging [5]. Lower extremity injuries (such as ACL tears and ankle sprains) are common in sports, and understanding knee joint kinematics during stepping and jumping activities is essential to optimize rehabilitation protocols to increase the effectiveness of their treatment [6, 7]. Three-dimensional (3D) assessment (3D knee valgus) is considered the gold standard measurement method for assessing the knee valgus angle and is evaluated through motion analysis. This defines the relative relationship between the femur and tibia. However, 2-dimensional (2D) knee valgus assessment methods are popular due to their low cost, ease of application, and ability to screen many participants [8, 9].

Prevention training programs in football focus on improving coordination, strength, and proprioception. They aim to protect against non-contact ACL injuries by optimizing athletes’ neuromuscular strategies in high-risk maneuvers such as landing, pivoting, and cutting [10, 11]. Various injury prevention programs have been developed, including neuromuscular training and Injury Prevention and Performance Enhancement (PEP) [12]. Successful programs include traditional stretching, strengthening, awareness of high-risk positions, technique modification, aerobic capacity, sport-specific quickness-agility, proprioception, balance, and many plyometric exercises [13]. The principles underlying all protective programs include proprioception exercises, core strength, neuromuscular training, muscle memory, correct technique, control of the entire body, and lower extremity positioning. These approaches can reduce the risks associated with dangerous positions if the athlete trains properly [14]. Minimizing the risk of injury or preventing potential injuries is the simplest and most cost-effective way to manage sports injuries. Injury prevention is possible by improving an athlete’s motor skills, muscle strength, coordination, flexibility, and postural control [15]. Hewett et al. [16] argued that a preventive program incorporating stretching, plyometric, and strength training can reduce the forces acting on the knee upon landing and valgus and varus stresses. In preventing traumatic injuries, identifying athletes at high risk of ACL injury is just as important as developing a preventive program. When designing a program, considering the appropriate timing for implementation is crucial, as it influences sports performance, muscle fatigue, skill development, and overall athletic ability [17]. Video analysis using the example of an anterior cruciate ligament (ACL) rupture was determined to show that the knee joint is most often (81%) in a valgus position during ACL rupture in professional men’s football [18]. Successful prevention programs typically include strength training, awareness of high-risk positions, traditional stretching, aerobic capacity development, technique modification, sport-specific agility training, balance exercises, proprioception drills, and various plyometric exercises [13]. Plyometric exercises such as jumping, hopping, and leaping are widely used to improve lower extremity performance through a stretch-shortening cycle [19]. An imbalance in muscle strength between the hamstrings and quadriceps is a known risk factor for non-contact ACL injuries [20]. Stretching exercises lengthen muscles and activate connective tissues [21]. Specifically, static stretching exercises are more effective than dynamic, PNF, or ballistic exercises in improving flexibility [22, 23]. Since flexibility exercises enhance joint range of motion and plyometric exercises improve strength, it is reasonable to expect that a training program designed to prevent ACL injuries will yield similar benefits [24].

While the literature supports ACL injuries as a primary contributing factor to sports-related injuries, it also highlights that football players frequently experience lower extremity injuries. Furthermore, the evaluation of intermuscular strength, balance, and flexibility in football players and the design of training programs to prevent injuries explain the importance of this study. These insights demonstrate the study’s contribution to the literature, emphasizing the need and importance of preventive programs to prevent injuries by strengthening the muscles around the knee and improving balance and movement control. This study found that combining flexibility, strength, and plyometric exercises significantly improves jumping performance and reduces knee valgus angle in football players. While Hewett [16] and Neilson et al. [25] emphasized the role of plyometric and landing-focused training in enhancing neuromuscular control and reducing ACL injury risk, the present study shows a broader impact through a multi-component approach. Compared to the proprioceptive-focused interventions by Kilic et al. [26] and Caraffa [27], this study provides a more comprehensive evaluation by integrating both performance and biomechanical measures. Additionally, the findings align with Herman et al. [28], who noted that strength training alone is insufficient, reinforcing the value of combined training. This research supports multi-component programs as effective strategies for enhancing performance and preventing injuries, particularly ACL injuries, by addressing key biomechanical risk factors.

Based on this information, the study hypotheses are as follows:

### H1

Supplementary training programs incorporating stretching, strength, and plyometric exercises will significantly improve the plyometric performance of football players compared to regular training alone.

### H1a

Supplementary training programs will significantly reduce knee valgus angles among football players, enhancing lower limb alignment and potentially decreasing injury risk.

### Research question

What is the effect of stretching, strength, and plyometric exercise training programs, in addition to regular training, on jumping performance and the development of knee valgus values in football players?

## Methods

### Participants

#### Study sample size determination

The a priori power analysis for a two-group repeated‐measures MANOVA (using Pillai’s trace criterion and the O’Brien–Shieh algorithm) indicates that, to detect a moderate within‐subjects effect size (f = 0.25) across three measurement occasions with α = 0.05 and desired power of 0.95, a total sample of 86 participants is required. Specifically, the analysis yielded a noncentrality parameter of λ = 16.125, corresponding to an F‐critical value of F(2, 83) = 3.1065. The numerator degrees of freedom (df₁ = 2) reflect the number of within‐subject contrasts (measurements– 1), while the denominator degrees of freedom (df₂ = 83) represent the residual error after accounting for group and time effects [29].

With 86 participants (43 per group, assuming equal allocation), the study achieves an actual power of 0.9513, slightly exceeding the target of 0.95. This ensures a high probability of detecting an actual time-by‐group interaction of moderate magnitude, even under the conservative assumption of zero correlation among repeated measures. In sum, enrolling 86 subjects will provide sufficient sensitivity to assess whether the treatment administered to the experimental group produces a statistically reliable change over time compared to the control group.

### Sample of study

This study employed purposive sampling to select participants who met specific criteria relevant to the research objectives. This non-probability sampling technique involves the deliberate selection of individuals based on their characteristics, expertise, or experience, which are deemed valuable for addressing the research question [30].

The sample of this study consisted of male football players from an under-19 (U-19) team based in Kayseri, Türkiye. The study was conducted at Erciyes University, Faculty of Sports Sciences. The inclusion criteria required participants to be male, aged 18 or older, actively competing in the U-19 category, free from any disabilities, and to have at least five years of experience as licensed football players. Ninety athletes from an amateur football club were invited to participate in the study in 2024. From an amateur football club in Kayseri, Türkiye, ninety athletes who fulfilled these criteria were invited to participate in the study. The selection was made in collaboration with team coaches based on club records confirming eligibility.

The selection of U-19 (18–19 years old) male football players as participants in this study is based on multifaceted scientific and practical considerations. This age group represents a critical transition period to a professional career, during which motor development and athletic performance reach advanced levels. Therefore, examining the effects of training programs during this phase yields findings that can directly influence players’ career development. Controlled studies involving athletes at this stage, nearing the completion of their growth and development, offer more reliable and generalizable results regarding the effectiveness of training interventions. Furthermore, the physiological and biomechanical responses observed in this group serve as a bridge between youth and adult athletes, enhancing the scientific value of the research. In conclusion, including U-19 football players in the study provides practical benefits in individual performance enhancement and injury prevention, while enabling the scientific investigation of age-specific training responses.

Participants were assigned to experimental and control groups using an unbiased sampling method. Participants were evenly distributed between the two groups based on their pre-test results to ensure group equivalence. The study employed a proper experimental design, specifically the pre-test–post-test control group model. This model is characterized by including multiple groups and the random assignment of participants [31]. In this design, both groups underwent measurements before and after the intervention. However, the training program was applied exclusively to the experimental group.

### Randomised method

The study included 90 male U-19 football players actively competing in clubs based in Kayseri. Using a simple randomization method, participants were randomly assigned to either the experimental group (*n* = 45) or the control group (*n* = 45). The random allocation sequence was generated by an independent statistician using computer-based software. Group assignments were implemented with sequentially numbered, opaque, sealed envelopes, and allocation concealment was maintained until the interventions were initiated.

Due to the interventions’ nature, blinding participants and implementers was not feasible. However, the data analyst responsible for outcome evaluation was blinded to group assignments. All analyses were conducted using coded labels (“Group A” and “Group B”) to ensure objectivity during the evaluation phase [32].

### Collection of data

#### Demographic tests

**Demographic Characteristics:** Participants’ height, age, body mass index (BMI), and body weight measurements were taken for scientific purposes only.

**Weight measurement: **The body weights of the football players were measured using an electronic Xiaomi MI Body Composition Scale 2 scale to an accuracy of ± 0.1 kg [33].

**Height measurement:** Height measurements of the participants were measured with a Fisco brand meter with an accuracy of ± 0.1 cm [34].

**BMI:** Body mass index values of the participants were calculated using the formula of the ratio of height squared to body weight (kg/m2) [35].

### Plyometric test and valgus tests

Jump tests of the participants (CMJ, CMJFREE, DJ, SJ, and Horizontal Jump) were performed with the My Jump 2 application [36].

#### My jump 2

My Jump 2 mobile application measures jump height with high-speed video recording. “My Jump 2”, an iPhone application used to calculate jump height, was initially developed with Xcode software [37–40]. Before the measurement, the participant’s leg length and body weight are determined and recorded in the application. The jumping movement of the participant is recorded through the application, and then the point where their feet leave the ground and the point where they touch the ground again are determined through the application. The application calculates the flight time (ms), jump height (cm), speed (m/s), force (N), and power (W) values of the jump [36]. During the research, an iPhone 15 model mobile phone was used to collect data through the My Jump 2 application. Ulupınar et al. [41] showed that analyzing bounce video recordings with the My Jump application offers high reliability within and between evaluators. My Jump 2 application’s reliability was high in 20 and 40 cm drop jump tests. ICC values ​​for RSI were calculated as 0.95 for 20 cm and 0.98 for 40 cm. Internal consistency (Cronbach’s Alpha) was 0.98 for 20 cm and 0.99 for 40 cm; CV was 6.71% for 20 cm and 10.32% for 40 cm. All parameters except power measurement showed high reliability. The results show that the application is a reliable tool for drop jump and RSI measurements [40].

#### Countermovement jump (CMJ) test

**Fig. 1:**
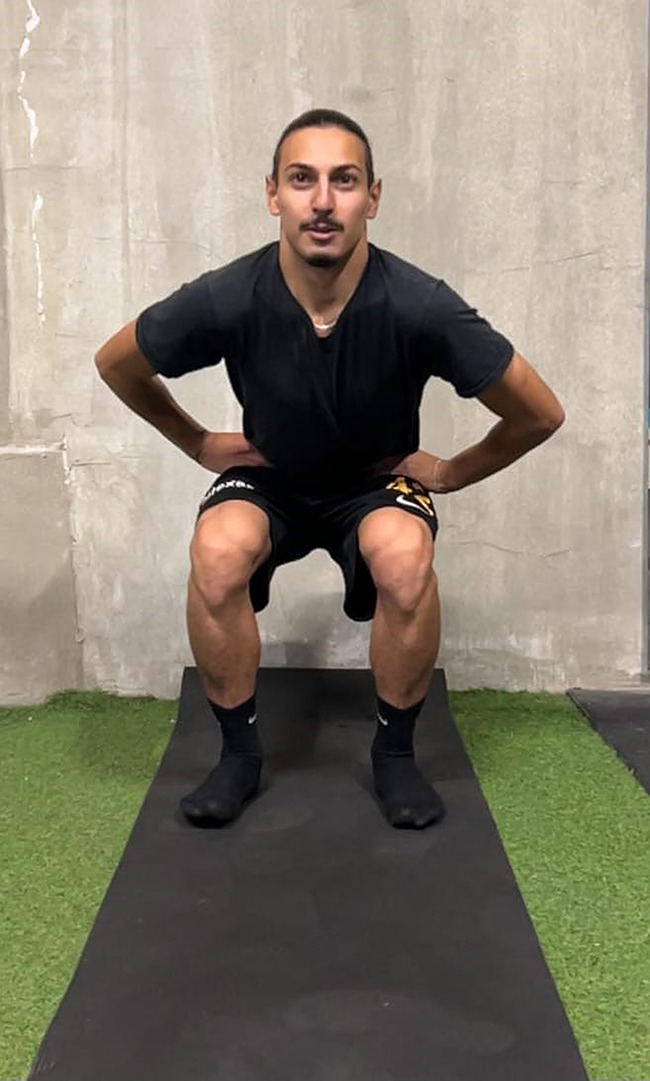
Countermovement jump (CMJ) test

In the widely used Countermovement Jump Test application, the hands remain on the hips throughout the test. When ready, after squatting, the subject immediately jumps as vertically as possible until his knees are 90 degrees, simultaneously landing back on both feet. A good rest is needed between attempts [42]. Countermovement jump (CMJ) performance is shown in Fig. 1.

#### Countermovement jump free (CMJ Free) test

**Fig. 2:**
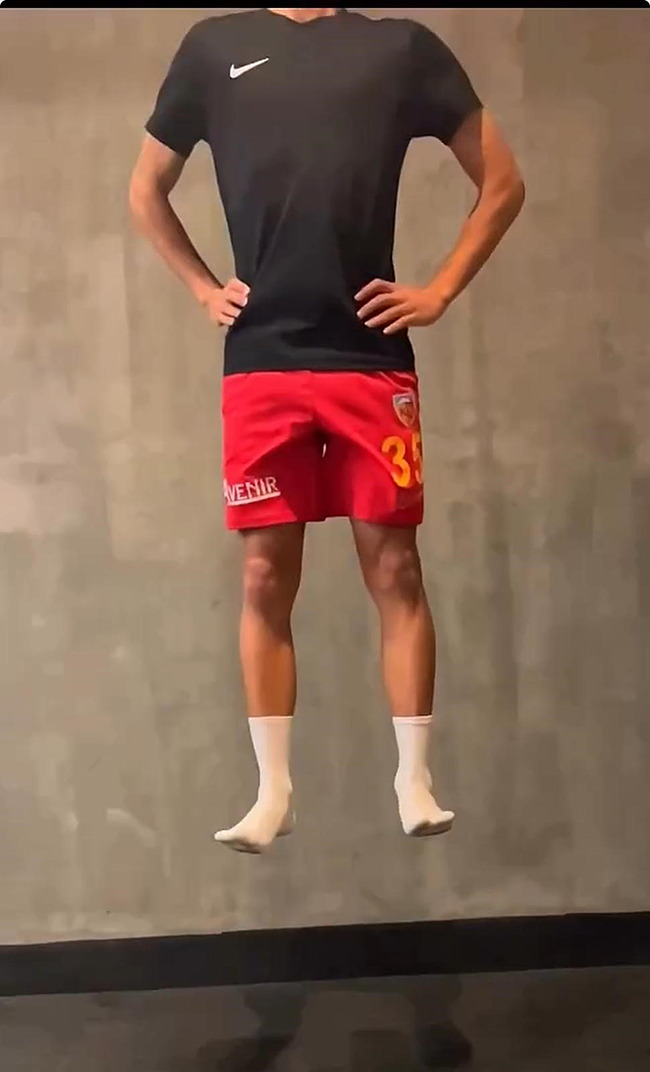
Countermovement jump free (CMJ Free) test

The only rule is that the participant reaches the maximum possible height when performing CMJ (CMJ free) free arm swing [43]. Countermovement jump free (CMJ Free) performance is shown in Fig. 2.

#### Drop jump test (DJ)

**Fig. 3:**
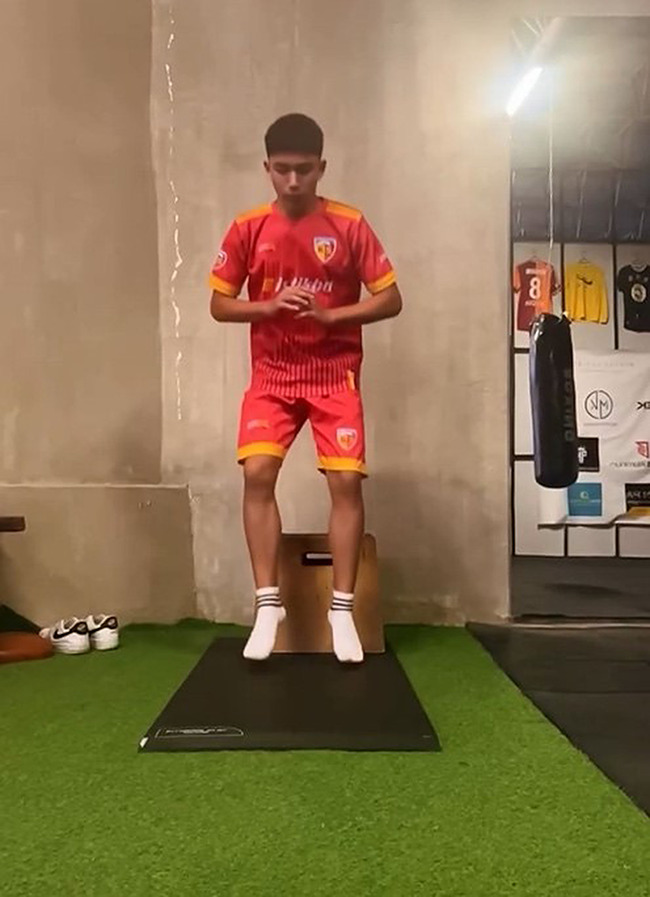
Drop jump test (DJ)

A jump test was applied to the participants from a height of 40 cm with three repetitions with their hands on their waist, and their values were recorded [44]. The drop jump test (DJ) performance is shown in Fig. 3.

#### Squat jump (SJ) test

**Fig. 4:**
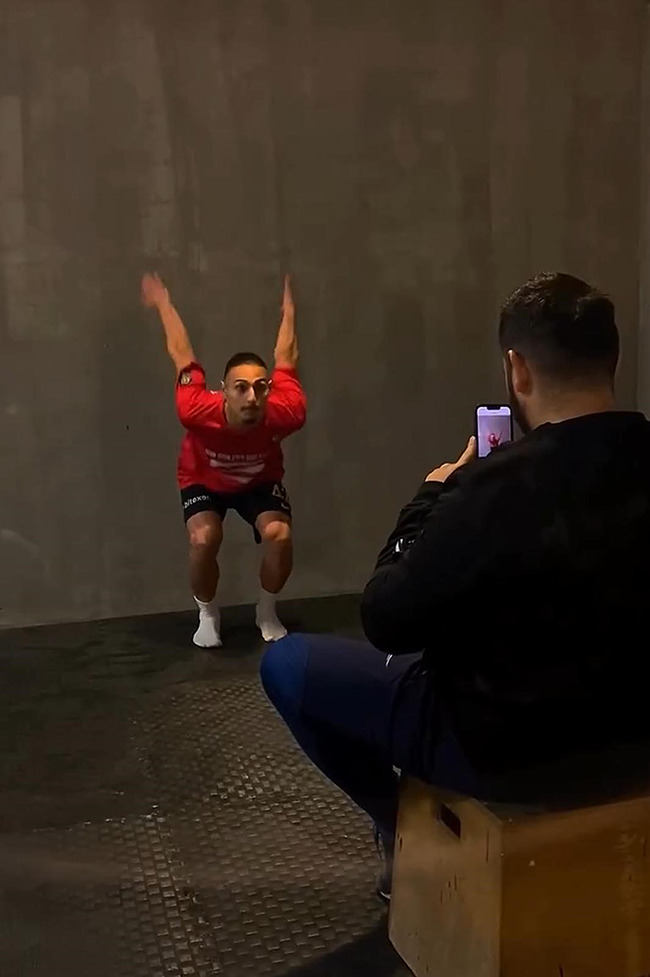
Squat jump (SJ) test

The Squat Jump Test begins by bending the knee joint and reaching a 90° angle in a stable position. After the bending, when the subject is ready, it is performed by opening the knee joint with the feet pushing the ground and gaining force from the ground. It is a test protocol in which the jumping force is measured vertically. Three attempts are given in the test, and the highest one is considered valid [45]. Squat jump (SJ) test performance is shown in Fig. 4.

#### Horizontal jump test

**Fig. 5:**
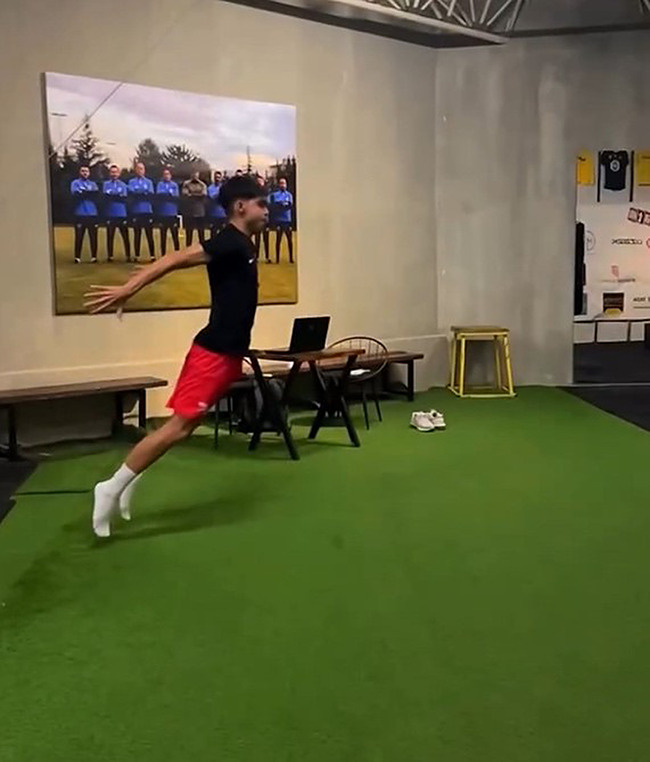
Horizontal jump test

Participants are asked to stand at the previously determined starting point. The measurement is carried out by recording the stage from the moment their feet leave the ground at the first point they start jumping, to the moment their feet first touch the ground when they jump, and the moment they land, with the My Jump 2 application [36]. Horizontal jump test performance is shown in Fig. 5.

#### Knee valgus test

**Fig. 6:**
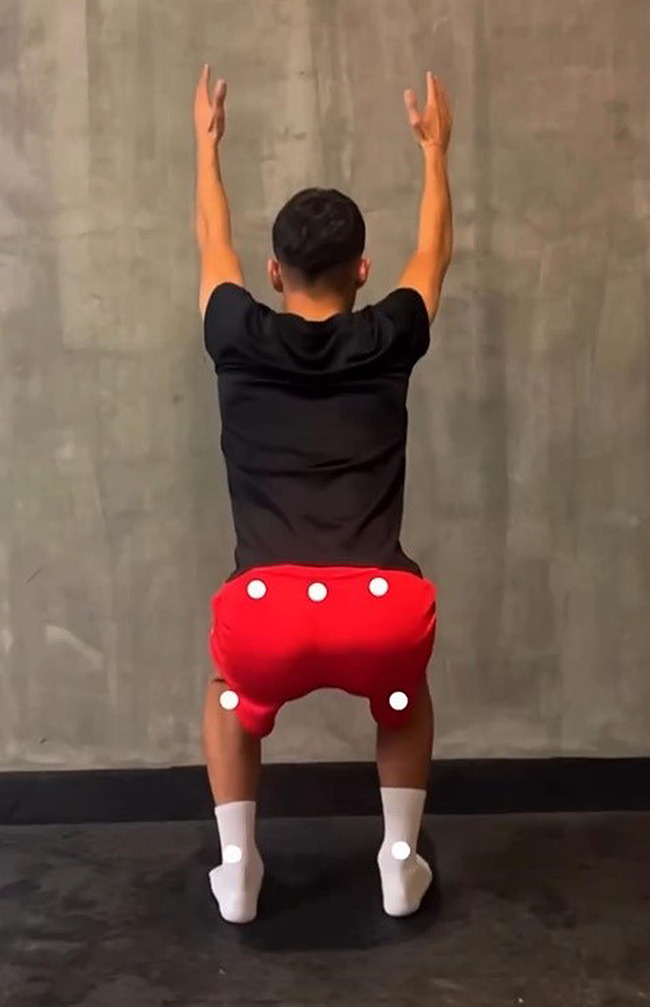
Knee valgus test

The knee valgus test was performed using the My Jump Lab app [35]. It allows us to perform motion analysis without the need for any markers. It offers the opportunity to evaluate knee valgus. It analyzes the image recorded by the phone. In line with the analyses, it provides important ideas about preventing injuries, especially in the lower extremities, and the ideal implementation of technical movements [36]. Knee valgus test performance is shown in Fig. 6.

#### Training programs

The training programs consisted of strength, flexibility, and plyometric exercises designed to reduce muscle strength imbalances in participants while enhancing their flexibility and plyometric performance. Since improving motor characteristics and achieving muscle balance are expected to impact valgus values positively, all training components were applied together. Strength training was implemented for eight weeks, including full-body, core, and leg exercises. Additionally, the Hewett et al. [16] anterior cruciate ligament injury prevention program, which focuses on plyometric exercises, was applied for the first six weeks, aligning with the program’s duration [16]. Stretching exercises were performed after strength training for the entire eight-week period. The training was administered exclusively to participants in the experimental group, following a consistent schedule and sequence. The program was conducted three days a week for eight weeks, separate from regular football training. Plyometric exercises were included only one day per week, and a minimum one-day rest period was maintained between training sessions.

**Table 1:** General exercise training program

Week	Day 1	Day 2	Day 3
Week 1	Core Exercise Training, Plyometric Exercise Training, Stretching Exercise Training	Leg Exercise Training, Stretching Exercise Training	Complete Body Exercise Training, Stretching Exercise Training
Week 2	Leg Exercise Training, Stretching Exercise Training	Complete Body Exercise Training, Stretching Exercise Training	Core Exercise Training, Plyometric Exercise Training, Stretching Exercise Training
Week 3	Complete Body Exercise Training, Stretching Exercise Training	Core Exercise Training, Plyometric Exercise Training, Stretching Exercise Training	Leg Exercise Training, Stretching Exercise Training
Week 4	Core Exercise Training, Plyometric Exercise Training, Stretching Exercise Training	Leg Exercise Training, Stretching Exercise Training	Complete Body Exercise Training, Stretching Exercise Training
Week 5	Leg Exercise Training, Stretching Exercise Training	Complete Body Exercise Training, Stretching Exercise Training	Core Exercise Training, Plyometric Exercise Training, Stretching Exercise Training
Week 6	Complete Body Exercise Training, Stretching Exercise Training	Core Exercise Training, Plyometric Exercise Training, Stretching Exercise Training	Leg Exercise Training, Stretching Exercise Training
Week 7	Core Exercise Training, Plyometric Exercise Training, Stretching Exercise Training	Leg Exercise Training, Stretching Exercise Training	Complete Body Exercise Training, Stretching Exercise Training
Week 8	Leg Exercise Training, Stretching Exercise Training	Complete Body Exercise Training, Stretching Exercise Training	Core Exercise Training, Plyometric Exercise Training, Stretching Exercise Training

Table 1 shows the details of the general exercise training program applied to the experimental group, 3 days a week for 8 weeks.

Strength exercise training program: Strength exercises include full body strength training, core exercise training, and leg exercise training designed by the researcher who conducted the study’s training. Before the strength training, the participants warmed up individually with jogging and stretching.

**Table 2:** Strength exercise training

Full Body Strength Training		Sets	
Chest press		3 × 12	
Butterfly		3 × 12	
Lat pull down		3 × 12	
Cable row		3 × 12	
Neck press machine		3 × 12	
Dumbbell side lateral raises		3 × 12	
Dumbbell curl		3 × 12	
Cable curl		3 × 12	
Push down		3 × 12	
Kickback		3 × 12	
Core Exercise Training		Time	Sets
Plank		40 s	3
Side plank		30 s	3
Lying hip raises		40 s	3
Bird dog		40 s	3
Oblique twist		30 s	3
Cobra exercise		30 s	3
Leg Exercise Training		Sets	
Leg press		4 × 12	
Smith machine squat		4 × 12	
Leg extension		4 × 12	
Leg curl		4 × 12	
Lunge dumbbell		4 × 12	
Calf raises		4 × 20	
Hamstring	Nordic curl	4 × 12	
	Single leg deadlift	4 × 12	
	Hip thrust	4 × 12	
	Theraband hamstring one leg	4 × 12	
	Box reverse lunges	4 × 12	

Table 2 shows the details of the strength training program applied to the participants in the experimental group.

**Table 3:** Anterior cruciate ligament injury prevention program [16, 46]

Plyometric Exercise Training Program		
Phase: 1 Technique	Week 1	Week 2
Wall jumps	20 s	25 s
Tuck jumps (use mat)	20 s	25 s
Broad jumps stick (hold) landing.	5 sets	10 sets
Squat jumps (use mat)	10 s	15 s
Double-legged cone jumps (use mat)	30 s/ 30 s	30 s/30sec
180-degree jumps	20 s	25 s
Bounding in place	20 s	25 s
Phase: 2 Technique	Week 3	Week 4
Wall jumps	30 s	30 s
Tuck jumps (use mat)	30 s	30 s
Jump, jump, jump, vertical jump	5 sets	8 sets
Squat jumps (use mat)	20 s	20 s
Bounding for distance	1 run	2 runs
Double-legged cone jumps (use mat)	30 s/ 30 s	30 s /30sec
Scissors jump	30 s	30 s
Hop, hop, stick landing (use mat)	5 sets/leg	5 sets/leg
Phase: 3 Technique	Week 5	Week 6
Wall jumps	30 s	30 s
Step, jump up, down, vertical	5 sets	10 sets
Mattress jumps	30 s / 30 s	30 s / 30 s
Single-legged jumps distance (use mat)	5 sets/leg	5 sets/leg
Squat jumps (use mat)	25 s	25 s
Jump into bounding (use mat)	3 runs	4 runs
Hop, hop, stick landing	5 sets/ leg	5 sets/ leg

Table 3 details the ACL injury prevention program administered to the experimental group participants.

#### Plyometric exercise training program

Hewett argued that the forces, valgus, and varus stresses on the knee after a fall can be reduced with a preventive program that includes stretching, plyometrics, and strengthening. This prevention program focuses on muscle imbalances that cause participants to get injured. Reducing the rate of contraction imbalance between the hamstrings and quadriceps reduces the load and stress on the anterior cruciate ligament, thus reducing the possibility of injury. The prevention program has three stages [16].

#### Stretching program

The Santa Monica Improving Injury Prevention Performance (PEP) program is an ACL injury prevention program. Its advantages are that the program takes 15–20 min, does not require much equipment, and can be applied throughout the season. The PEP program consists of five main parts: warm-up, strengthening, stretching, plyometric training, and agility [12]. Stretching exercises were included in our study.

**Table 4:** PEP anterior cruciate ligament injury prevention program [46]

Stretching Exercise Training Program
Calf Stretch
Quadriceps Stretch
Hamstring Stretch
Inner Thigh Stretch
Hip Flexor Stretch

Table 4 details the PEP ACL injury prevention program administered to the experimental group.

### Analysis of data

The data used in the study were analyzed using the SPSS 27 program. The Kolmogorov-Smirnov test was performed to assess the normality of the data distribution. To examine the differences between groups, the Independent Samples T-test was used, as the sample consisted of two groups: experimental and control. The relationship between the post-test values ​​of the groups was analyzed using Pearson Correlation analysis. Repeated measures analysis was conducted to compare the pre-test, interim, and post-test values collected at three different time points, and since three measurements were involved, analysis of variance (ANOVA) was employed. A group-time interaction test was applied to compare the changes in pre-test, mid-test and post-test scores of the groups over time. n² was interpreted according to Cohen [47] and Richardson [48]. Additionally, the Bonferroni test was applied to identify the source of any significant differences between the measurements. In the present study, the group size was *n* = 45 per group, which exceeds the threshold where overly conservative correction procedures such as Bonferroni might significantly distort the Type I error control [49].

### Ethical statement

The study was approved by the Erciyes University Health Sciences Institute Social and Human Sciences Ethics Committee (367; approval date: 27.08.2024). Informed written consent was obtained from all subjects involved in the study.

## Results

**Fig. 7:**
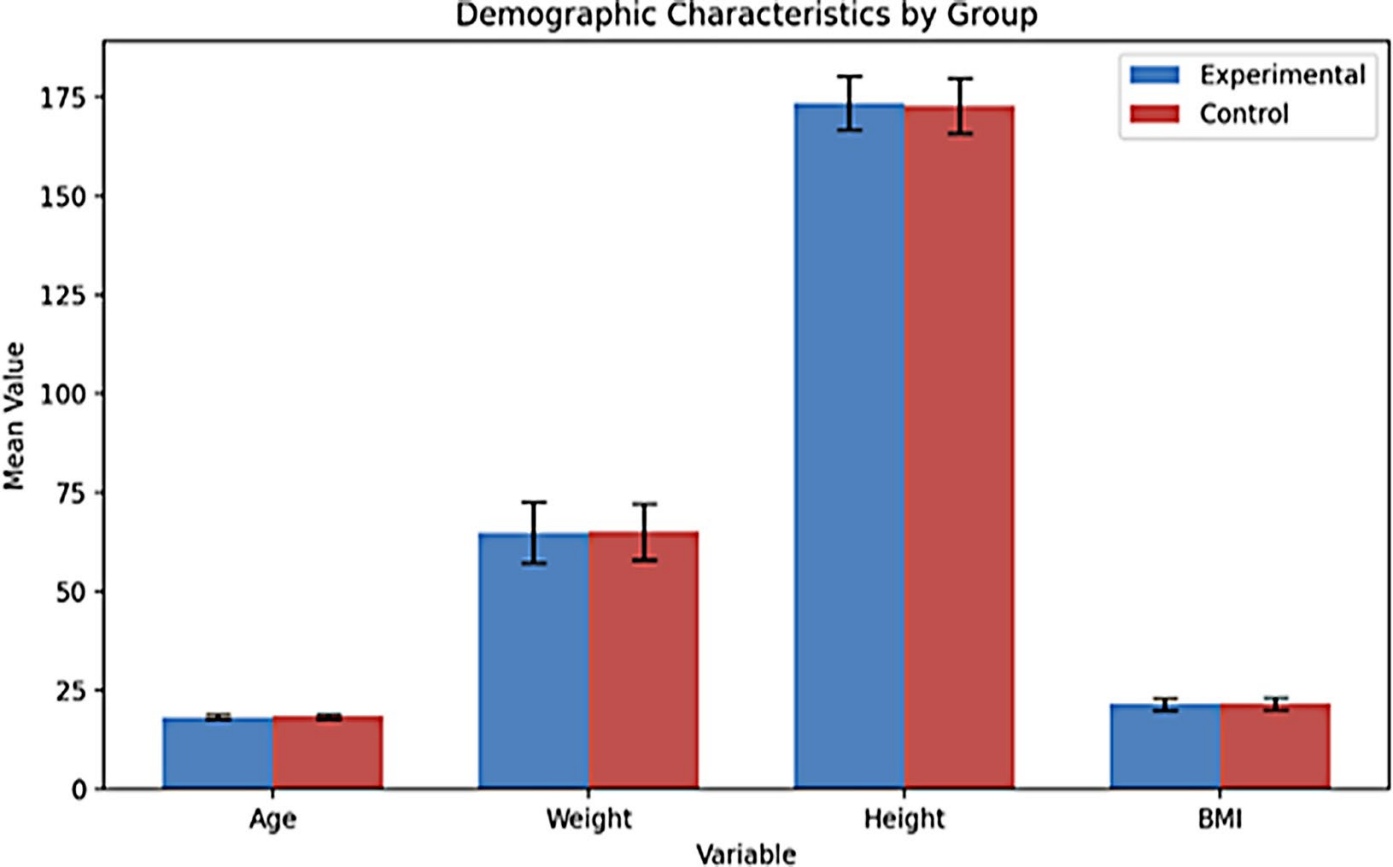
Graph of demographic information (age, weight, height, and BMI) of participants

Table 5 includes demographic information about the participants. When the average age of the participants is examined, the average for the experimental group is 18.42, and the average for the control group is 18.44. The average weight of the experimental group is 64.95, and the average for the control group is 65.13. When the participants’ height is examined, it is 173.51 for the experimental group and 172.86 for the control group. When the average BMI is examined, it is determined that the experimental group is 21.50, and the control group is 21.75. Figure 7 shows the graph of the demographic information data (age, weight, height, BMI) of the participants.

**Table 5:** Demographic information

Variable	Group	*N*	Minimum	Maximum	x̄	SD
Age	Experimental	45	18.00	19.00	18.42	0.49
	Control	45	18.00	19.00	18.44	0.50
Weight	Experimental	45	49.00	80.00	64.95	7.74
	Control	45	50.00	80.00	65.13	7.10
Height	Experimental	45	160.00	188.00	173.51	6.77
	Control	45	161.00	189.00	172.86	7.01
BMI	Experimental	45	19.05	24.96	21.50	1.50
	Control	45	19.11	26.99	21.75	1.46

**Table 6:** Comparison of jump pre-test and post-tests between groups

Test		Group	*N*	x̄	SD	t	df	*p*	Cohen’s d
CMJ	Pre	Experimental	45	37.13	5.93	0.024	88	0.981	
		Control	45	37.10	5.85				
	Post	Experimental	45	41.78	5.39	2.283	88	0.025*	0.481
		Control	45	39.16	5.49				
CMJ FREE	Pre	Experimental	45	43.79	7.46	0.078	88	0.938	
		Control	45	43.67	7.23				
	Post	Experimental	45	49.10	6.76	2.170	88	0.033*	0.458
		Control	45	45.98	6.87				
DJ	Pre	Experimental	45	39.71	6.52	− 0.168	88	0.867	
		Control	45	39.94	6.37				
	Post	Experimental	45	46.42	6.40	2.267	88	0.026*	0.478
		Control	45	43.37	6.35				
SJ	Pre	Experimental	45	36.78	6.76	0.050	88	0.960	
		Control	45	36.71	6.69				
	Post	Experimental	45	42.13	6.02	2.221	88	0.029*	0.468
		Control	45	39.22	6.39				
H. JUMP	Pre	Experimental	45	171.74	21.20	0.004	88	0.997	
		Control	45	171.72	20.78				
	Post	Experimental	45	186.54	14.72	2.902	88	0.005**	0.612
		Control	45	175.53	20.76				

Note. *p <.05, **p <.01, Cohen’s d = 0.20 = Small effect, 0.50 = Medium effect, 0.80 or higher = Large effect

**Fig. 8:**
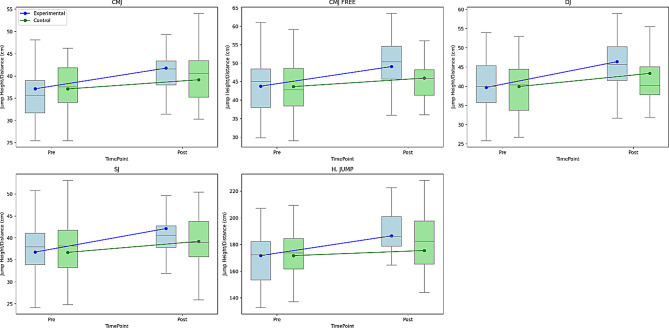
Pre-test and post-test comparisons of jump performance between experimental and control groups across different jump types. (**A**) CMJ – Countermovement Jump, (**B**) CMJ FREE – Free Arm Countermovement Jump, (**C**) DJ – Drop Jump, (**D**) SJ – Squat Jump, (**E**) H. JUMP – Horizontal Jump. Jump height/distance is measured in centimeters (cm). Lines represent group means across time points

Table 6 presents the pre- and post-test results for various jump performance tests (CMJ, CMJ FREE, DJ, SJ, and Horizontal Jump) in both the experimental and control groups. At baseline, no significant differences existed between groups in any of the tests (*p* >.05). Following the intervention, the experimental group demonstrated statistically significant improvements in all jump tests compared to the control group, as indicated by the post-test means and p-values (*p* <.05 for all). Effect sizes (Cohen’s d) were calculated as follows: a small effect for CMJ (d = 0.481), CMJ Free (d = 0.458), DJ (d = 0.478), and SJ (d = 0.468), and a medium effect for horizontal jump (d = 0.612), with the most notable improvement observed in horizontal jump performance. These results suggest that the intervention effectively enhanced lower extremity explosive power and jumping performance in the experimental group, whereas only minimal changes occurred in the control group. Figure 8 shows the graph of the data comparing the pre-test and post-test values of different jump tests.

**Table 7:** Comparison of valgus pre-test and post-tests between groups

		Group	*N*	x̄	SD	t	df	*p*	Cohen’s d
RIGHT KNEE VALGUS	Pre	Experimental	45	4.55	3.12	0.137	88	0.891	
		Control	45	4.47	2.70				
	Post	Experimental	45	2.36	1.44	-3.392	88	0.001**	0.715
		Control	45	3.64	2.06				
LEFT KNEE VALGUS	Pre	Experimental	45	4.48	2.17	0.371	88	0.712	
		Control	45	4.32	1.96				
	Post	Experimental	45	2.58	1.36	-4.607	88	0.001**	0.971
		Control	45	4.20	1.92				

Note. **p <.01, Cohen’s d = 0.20 = Small effect, 0.50 = Medium effect, 0.80 or higher = Large effect

**Fig. 9:**
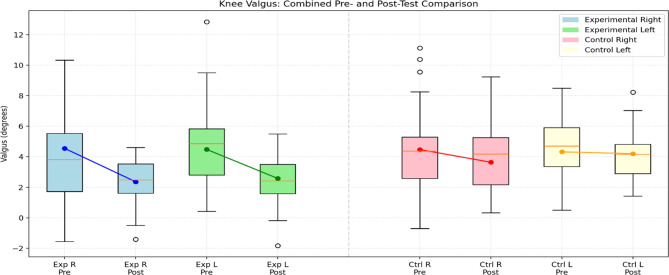
Comparison of pre-test and post-test knee valgus angles (in degrees) between experimental and control groups. (Exp R: Experimental Right, Exp L: Experimental Left, Ctrl R: Control Right, Ctrl L: Control Left). The lines indicate the mean changes across conditions

Table 7 presents the pre- and post-test results for various knee valgus tests (Right Knee Valgus, Left Knee Valgus) in both the experimental and control groups. At baseline, no significant differences existed between groups in any of the tests (*p* >.05). Following the intervention, the experimental group showed statistically significant improvements in both valgus tests compared to the control group, as indicated by the post-test means and p-values (*p* <.05 for all). The effect sizes (Cohen’s d) were large for right knee valgus (d = 0.715) and left knee valgus (d = 0.971), with the most significant improvement observed for left knee valgus (Cohen’s d = 0.971). These effect sizes show that the difference between the test values between the groups is large.These findings show that the intervention improved knee valgus in the experimental group, while the control group showed minimal changes. Figure 9 shows the graph comparing the participants’ knee valgus.

**Table 8:** Comparison within the group for the CMJ

Variable	Group	Test	x̄ ± SD	X^2^	Groups			Time			Group*Time			Bonferoni
					F	p	η^2^	F	p	η^2^	F	p	η^2^	
CMJ	Experimental	Pre	37.13 ± 5.93	32.735	89.651	0.000 **	0.671	162.152	0.000 **	0.648	25.021	0.000 **	0.221	1 < 2 1 < 3 2 < 3
		Mid	39.18 ± 5.63											
		Post	41.78 ± 5.39											
	Control	Pre	37.10 ± 5.85		120.393	0.000 **	0.732							1 < 2 1 < 3 2 < 3
		Mid	38.29 ± 5.68											
		Post	39.16 ± 5.49											

Note. **p <.01, n^2^ = 0.01 = Small effect, *p* =.06, = Medium effect, *p* =.14 Large effect. 1 = Pre-test, 2 = Mid-test, 3 = Post-test

When Table 8 is examined, the results of the repeated measures ANOVA regarding CMJ performance show that there are significant differences over time in both the experimental and control groups. In the experimental group, a significant difference was found between the pre-test (𝑥̄ = 37.13 ± 5.93), mid-test (𝑥̄ = 39.18 ± 5.63), and post-test (𝑥̄ = 41.78 ± 5.39) measurements [F(2, X) = 89.651, *p* <.001, η² = 0.671]. This effect size is considered large (η² >0.14) and (η² = 0.671), indicating that the experimental group showed significant improvement in CMJ performance with the applied intervention.

Similarly, in the control group, significant differences were found between the pre-test (𝑥̄ = 37.10 ± 5.85), mid-test (𝑥̄ = 38.29 ± 5.68), and post-test (𝑥̄ = 39.16 ± 5.49) [F(2, X) = 120.393, *p* <.001, η² = 0.732]. This effect size also indicates a large effect. However, the average development values were lower compared to the experimental group.

When the main effect of time is examined, it is evident that all participants showed significant and high-level improvement in CMJ performance over time [F = 162.152, *p* <.001, η² = 0.648]. This effect size is also large, showing that the time factor has a strong impact on the entire sample.

The most critical finding is that the interaction between group and time is statistically significant and large [F = 25.021, *p* <.001, η² = 0.221]. This finding indicates that the changes in the experimental and control groups over time differ, and that the intervention applied to the experimental group had a significantly higher effect on CMJ performance than the control group. The effect size (η² = 0.221) shows that the group-time interaction also has a large effect.

**Fig. 10:**
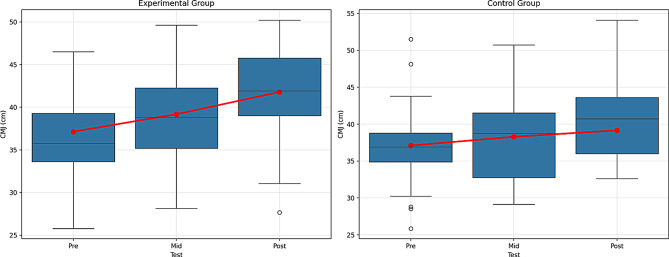
Pre-test, mid-test, and post-test comparisons of countermovement jump (CMJ) performance in (**A**) the experimental group and (**B**) the control group. Red lines represent group mean values. CMJ performance is expressed in centimeters (cm)

According to the Bonferroni multiple comparison test results, significant differences were found between the pre-test, mid-test, and post-test measurements in both the experimental and control groups (1 < 2, 1 < 3, 2 < 3). However, the higher average increase in the experimental group compared to the control group clearly supports the effectiveness of the applied program. Figure 10 shows the graph of the within-group comparison of the pre-test, mid-test, and post-test values of the CMJ tests applied to the experimental and control groups.

**Table 9:** Comparison within the group for the CMJ free

Variable	Group	Test	x̄ ± SD	X^2^	Group			Time			Grup*Time			Bonferroni
					F	p	η^2^	F	p	η^2^	F	p	η^2^	
CMJ Free	Experimental	Pre	43.79 ± 7.46	21.801	80.897	0.000**	0.648	138.007	0.000**	0.611	21.129	0.000 **	0.194	1 < 2 1 < 3 2 < 3
		Mid	46.11 ± 6.74											
		Post	49.10 ± 6.76											
	Control	Pre	43.67 ± 7.23		73.312	0.000**	0.625							1 < 2 1 < 3 2 < 3
		Mid	44.55 ± 7.13											
		Post	45.98 ± 6.87											

Note. **p <.01, n^2^ = 0.01 = Small effect, *p* =.06, = Medium effect, *p* =.14 Large effect. 1 = Pre-test, 2 = Mid-test, 3 = Post-test

According to the Table 9, the experimental group showed significant improvement over time. The differences between the pre-test (43.79 ± 7.46), mid-test (46.11 ± 6.74), and post-test (49.10 ± 6.76) values were found to be statistically significant (F = 80.897, *p* <.01). This improvement shows a very large effect when the effect size (η² = 0.648) is considered. In other words, the change experienced in the experimental group over time is substantial, and the effect of the intervention is strong. This finding suggests that the intervention was effective in improving CMJ Free performance.

The control group also showed some changes over time. Significant differences were observed between the pre-test (43.67 ± 7.23), mid-test (44.55 ± 7.13), and post-test (45.98 ± 6.87) (F = 73.312, *p* <.01). The effect size (η² = 0.625) is large. Although improvement was seen in the control group over time, this change has a smaller effect size compared to the experimental group. This indicates that the improvement in the control group was less pronounced, and their performance increase was more limited, as they were not exposed to the intervention.

The Group × Time interaction is also significant (F = 138.007, *p* <.01). This interaction indicates that both groups changed over time, but at different rates. The effect size (η² = 0.194) is at a large effect level. The experimental group showed faster and more pronounced improvement due to the intervention, while the control group showed only limited improvement over time.

**Fig. 11:**
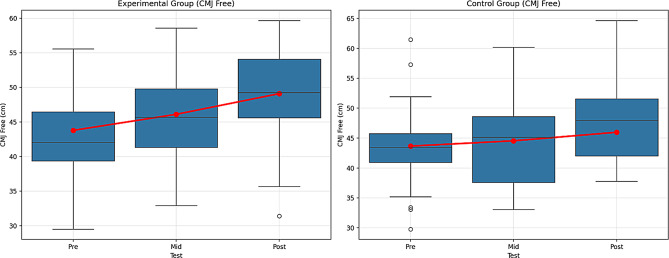
Pre-test, mid-test, and post-test comparisons of countermovement jump free (CMJ Free) performance in (A) the experimental group and (B) the control group. Red lines represent group mean values. CMJ Free performance is expressed in centimeters (cm)

Comparisons made with the Bonferroni correction reveal significant differences between the pre-test, mid-test, and post-test for both groups (Experimental group: 1 < 2, 1 < 3, 2 < 3; Control group: 1 < 2, 1 < 3, 2 < 3). However, there is a large difference between the post-test values of the two groups, with this difference being more pronounced in favor of the experimental group. Figure 11 shows the graph of the within-group comparison of the pre-test, mid-test, and post-test values of the CMJ Free tests applied to the experimental and control groups.

**Table 10:** Comparison within the group for the DJ

Variable	Group	Test	x̄ ± SD	X^2^	Group			Time			Grup*Time			Bonferroni
					F	p	η^2^	F	p	η^2^	F	p	η^2^	
DJ	Experimental	Pre	39.71 ± 6.52	94.454	121.726	0.000**	0.735	214.291	0.000 **	0.709	22.524	0.000 **	0.204	1 < 2 1 < 3 2 < 3
		Mid	42.95 ± 6.62											
		Post	46.42 ± 6.40											
	Control	Pre	39.94 ± 6.37		107.228	0.000**	0.709							1 < 2 1 < 3 2 < 3
		Mid	41.74 ± 6.48											
		Post	43.37 ± 6.35											

Note. **p <.01, n^2^ = 0.01 = Small effect, *p* =.06, = Medium effect, *p* =.14 Large effect. 1 = Pre-test, 2 = Mid-test, 3 = Post-test

According to the results in Table 10, the experimental group showed significant improvement over time. The differences between the pre-test (39.71 ± 6.52), mid-test (42.95 ± 6.62), and post-test (46.42 ± 6.40) were statistically significant (F = 121.726, *p* <.01). The effect size (η² = 0.735) was quite large, indicating that the improvement in the experimental group was substantial and that the effect of the intervention was highly significant. The increasing performance of the experimental group over time suggests that the intervention was effective and that this group experienced significant improvements on the CMJ Free test.

The control group also showed significant improvement over time. The differences between the pre-test (39.94 ± 6.37), mid-test (41.74 ± 6.48), and post-test (43.37 ± 6.35) were statistically significant (F = 107.228, *p* <.01). The effect size (η² = 0.709) is large, indicating improvement in the control group as well. However, since the effect size is slightly lower than that of the experimental group, this improvement is more limited and less pronounced. This suggests that the performance gains in the control group were constrained due to the lack of intervention.

The group and time interaction is also significant (F = 214.291, *p* <.01). This indicates that both groups changed over time, with differences in the rate of improvement between the two groups. The large interaction effect (η² = 0.709) suggests that the changes in both groups were significant, but the experimental group showed faster and greater improvement over time.

**Fig. 12:**
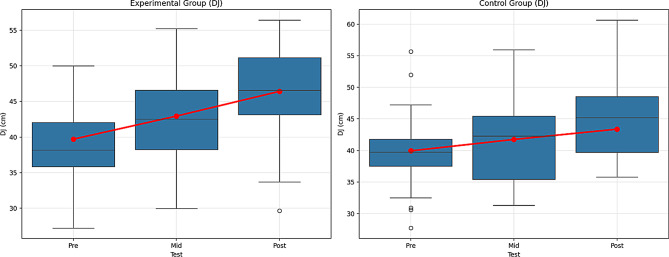
Pre-test, mid-test, and post-test comparisons of Drop Jump (DJ) performance in (**A**) the experimental group and (**B**) the control group. Red lines represent group mean values. DJ performance is expressed in centimeters (cm)

In the comparisons made with the Bonferroni correction, significant differences were found between the pre-test, mid-test, and post-test for both groups (Experimental group: 1 < 2, 1 < 3, 2 < 3; Control group: 1 < 2, 1 < 3, 2 < 3). The experimental group showed more substantial development over time, with the post-test value being higher than that of the control group. Figure 12 shows the graph of the within-group comparison of the pre-test, interim-test, and post-test values of the Drop Jump (DJ) tests applied to the experimental and control groups.

**Table 11:** Comparison within the group for the SJ

Variable	Group	Test	x̄ ± SD	X^2^	Group			Time			Grup*Time			Bonferroni
					F	p	η^2^	F	p	η^2^	F	p	η^2^	
SJ	Experimental	Pre	36.78 ± 6.76	42.234	110.433	0.000**	0.715	194.955	0.000**	0.689	25.957	0.000 **	0.228	1 < 2 1 < 3 2 < 3
		Mid	39.39 ± 6.30											
		Post	42.13 ± 6.02											
	Control	Pre	36.71 ± 6.69		110.557	0.000**	0.715							1 < 2 1 < 3 2 < 3
		Mid	38.23 ± 6.39											
		Post	39.22 ± 6.39											

Note. **p <.01, n^2^ = 0.01 = Small effect, *p* =.06, = Medium effect, *p* =.14 Large effect. 1 = Pre-test, 2 = Mid-test, 3 = Post-test

According to Table 11, the experimental group showed significant improvement over time. The differences between the pre-test (36.78 ± 6.76), mid-test (39.39 ± 6.30), and post-test (42.13 ± 6.02) were found to be statistically significant (F = 110.433, *p* <.01). The effect size (η² = 0.715) was quite large, indicating that the improvement in the experimental group was substantial, and the effect of the intervention was significant. The increasing performance over time suggests that the intervention was effective and that this group experienced significant improvements on the SJ test.

The control group also showed significant improvement over time. The differences between the pre-test (36.71 ± 6.69), mid-test (38.23 ± 6.39), and post-test (39.22 ± 6.39) were also statistically significant (F = 110.557, *p* <.01). The effect size (η² = 0.715) is again large, and improvement was observed in the control group as well. However, although the effect size is close to that of the experimental group, the improvement in the control group occurred without any intervention, meaning the effect of the intervention is limited.

The Group × Time interaction is also significant (F = 194.955, *p* <.01). This interaction reveals that both groups showed change over time, but these changes occurred at different rates and magnitudes. The large interaction effect (η² = 0.689) indicates that both groups improved over time, and the effects of these changes were significant. However, the experimental group showed faster and more pronounced improvement.

**Fig. 13:**
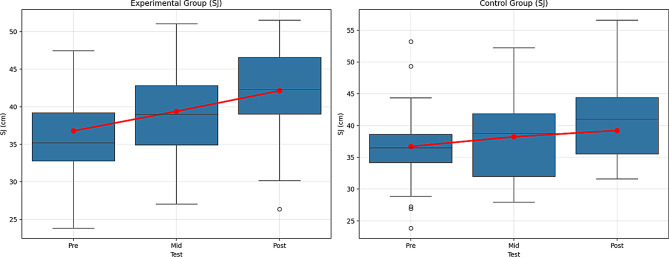
Pre-test, mid-test, and post-test comparisons of Squat Jump (SJ) performance in (**A**) the experimental group and (**B**) the control group. Red lines represent group mean values. SJ performance is expressed in centimeters (cm)

In the comparisons made with the Bonferroni correction, significant differences were found between the pre-test, mid-test, and post-test for both groups (Experimental group: 1 < 2, 1 < 3, 2 < 3; Control group: 1 < 2, 1 < 3, 2 < 3). The experimental group showed more significant improvement over time, with the post-test value being higher than the post-test value of the control group. Figure 13 shows the graph of the within-group comparison of the pre-test, mid-test, and post-test values of the Squat Jump (SJ) tests applied to the experimental and control groups.

**Table 12:** Comparison within the group for the horizontal jump

Variable	Group	Test	x̄ ± SD	X^2^	Group			Time			Grup*Time			Bonferroni
					F	p	η^2^	F	p	η^2^	F	p	η^2^	
Horizontal Jump	Experimental	Pre	171.74 ± 21.20	44.164	19.350	0.000**	0.305	29.667	0.000**	0.252	11.297	0.000**	0.114	1 < 2 1 < 3
		Mid	182.56 ± 15.60											
		Post	186.54 ± 14.72											
	Control	Pre	171.72 ± 20.78		382.831	0.000**	0.897							1 < 2 1 < 3 2 < 3
		Mid	173.52 ± 20.77											
		Post	175.53 ± 20.76											

Note. **p <.01, n^2^ = 0.01 = Small effect, *p* =.06, = Medium effect, *p* =.14 Large effect. 1 = Pre-test, 2 = Mid-test, 3 = Post-test

In Table 12, the experimental group showed a significant improvement between the pre- and mid-tests, with F = 19.350, *p* =.000 (effect size: 0.305, large effect), and between the pre- and post-tests, with F = 44.164, *p* =.000 (effect size: 0.252, large effect). These findings indicate a significant improvement in Horizontal Jump performance in the experimental group.

For the control group, the difference between the pre- and mid-tests was also found to be significant, with F = 382.831, *p* =.000 (effect size: 0.897, large effect). The difference between the pre- and post-tests was significant as well, with F = 29.667, *p* =.000 (effect size: 0.252, large effect), and the difference between the mid- and post-tests was significant with F = 11.297, *p* =.000 (effect size: 0.114, medium effect). These results show that there was an improvement in performance in the control group, but the effect size was smaller than that of the experimental group. While a significant increase in performance was observed over time for the experimental group, the control group also showed improvement, although the effect size was smaller.

**Fig. 14:**
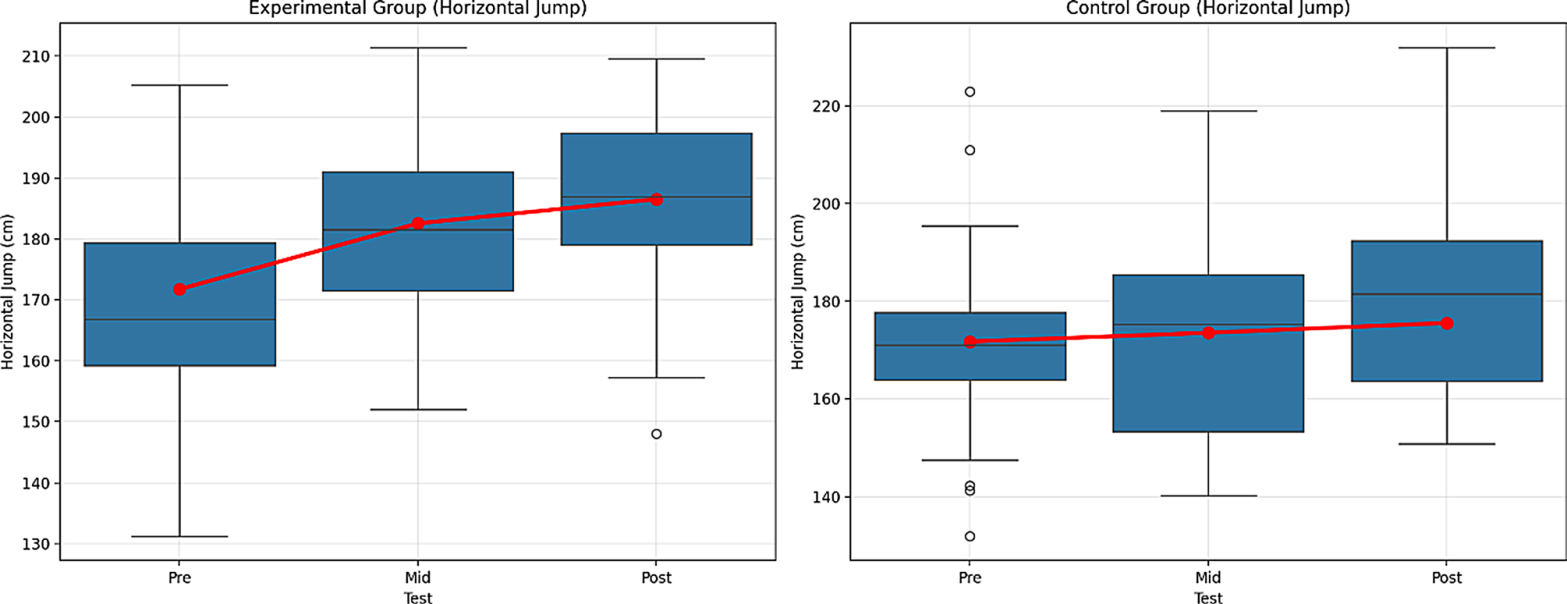
Pre-test, mid-test, and post-test comparisons of Horizontal Jump (HJ) performance in (**A**) the experimental group and (**B**) the control group. Red lines represent group mean values. SJ performance is expressed in centimeters (cm)

The Bonferroni test revealed significant differences for the experimental group between the pre- and mid-tests (1 < 2), and between the pre- and post-tests (1 < 3). For the control group, significant differences were found between the pre- and mid-tests (1 < 2), the pre- and post-tests (1 < 3), and the mid- and post-tests (2 < 3). Figure 14 shows the graph of the within-group comparison of the pre-test, interim-test, and post-test values of the Horizontal Jump tests applied to the experimental and control groups.

**Table 13:** Comparison within the group for the right knee valgus

Variable	Group	Test	x̄ ± SD	X^2^	Group			Time			Grup*Time			Bonferroni
					F	p	η^2^	F	p	η^2^	F	p	η^2^	
Right Knee Valgus	Experimental	Pre	4.55 ± 3.12	65.842	27.522	0.000**	0.385	41.653	0.000**	0.321	8.389	0.002*	0.087	1 > 2 1 > 3 2 > 3
		Mid	3.12 ± 2.05											
		Post	2.36 ± 1.44											
	Control	Pre	4.47 ± 2.70		15.372	0.000**	0.259							1 > 2 1 > 3 2 > 3
		Mid	3.92 ± 2.22											
		Post	3.64 ± 2.06											

Note. **p <.01, n^2^ = 0.01 = Small effect, *p* =.06, = Medium effect, *p* =.14 Large effect. 1 = Pre-test, 2 = Mid-test, 3 = Post-test

The findings in Table 13 examine the changes in Right Knee Valgus by comparing the experimental and control groups across different test times. The results of the Repeated Measures Analysis show that there are significant changes over time for both groups. The differences between the pre-, mid-, and post-tests in the experimental group were significant, with F = 27.522, *p* =.000 (large effect, η² = 0.385) and F = 65.842, *p* =.000 (large effect, η² = 0.321), respectively. These results demonstrate that the experimental intervention led to a significant improvement in Right Knee Valgus.

In the control group, the difference between the pre- and mid-tests was found to be significant, with F = 15.372, *p* =.000 (large effect, η² = 0.259), and the difference between the pre- and post-tests was significant, with F = 41.653, *p* =.000 (large effect, η² = 0.321). While an improvement was also observed in the control group, the effect size was smaller.

The Group × Time interaction analysis revealed that the performance changes of both groups were significantly different (F = 8.389, *p* =.002, small effect, η² = 0.087). The experimental group showed a stronger improvement in Right Knee Valgus, whereas the effect in the control group was smaller.

**Fig. 15:**
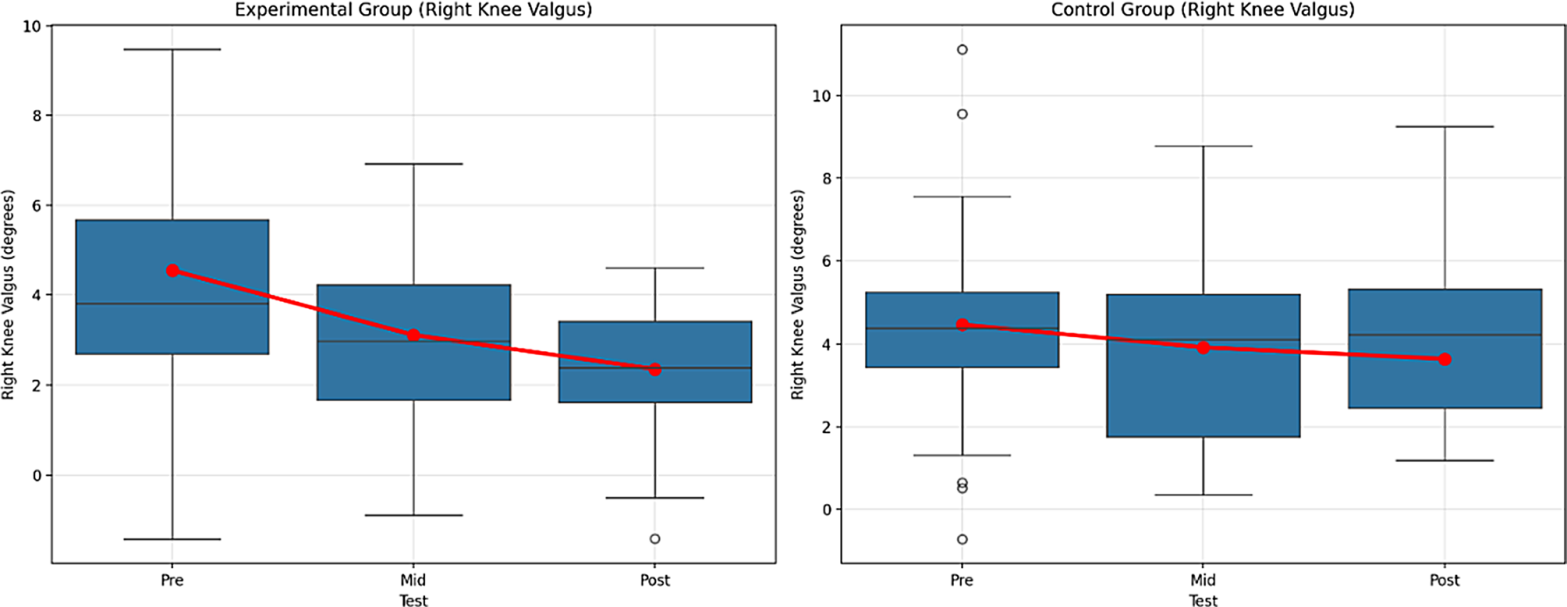
Pre-test, mid-test, and post-test comparisons of Right Knee Valgus performance in (**A**) the experimental group and (**B**) the control group. Red lines represent group mean values

According to the Bonferroni test results, significant differences were found between the times for both groups. For the experimental group, the differences between the pre- and mid-tests (1 > 2), pre- and post-tests (1 > 3), and mid- and post-tests (2 > 3) were significant. For the control group, significant differences were found between the pre- and mid-tests (1 > 2), pre- and post-tests (1 > 3), and mid- and post-tests (2 > 3). Figure 15 shows the graph of the within-group comparison of the pre-test, mid-test, and post-test values of the Right Knee Valgus tests applied to the experimental and control groups.

**Table 14:** Comparison within the group for the left knee valgus

Variable	Group	Test	x̄ ± SD	X^2^	Group			Time			Grup*Time			Bonferroni
					F	p	η^2^	F	p	η^2^	F	p	η^2^	
Left Knee Valgus	Experimental	Pre	4.48 ± 2.17	14.131	56.514	0.000**	0.562	56.439	0.000**	0.391	43.589	0.000**	0.331	1 > 2 1 > 3 2 > 3
		Mid	3.43 ± 1.72											
		Post	2.58 ± 1.36											
	Control	Pre	4.32 ± 1.96		1.816	0.169	-							-
		Mid	4.27 ± 1.87											
		Post	4.20 ± 1.92											

Note. **p <.01, n^2^ = 0.01 = Small effect, *p* =.06, = Medium effect, *p* =.14 Large effect. 1 = Pre-test, 2 = Mid-test, 3 = Post-test

The findings in Table 14 compare the changes in Left Knee Valgus in the experimental and control groups across different test times. According to the Repeated Measures Analysis, for the experimental group, the differences between the pre, mid, and post-tests were significant. The difference between the pre and mid-tests was significant, with F = 56.514, *p* =.000 (large effect, η² = 0.562), and the difference between the pre and post-tests was also significant, with F = 14.131, *p* =.000 (large effect, η² = 0.391). These findings show that the experimental intervention led to a significant improvement in Left Knee Valgus.

In the control group, the difference between the pre- and mid-tests was not significant (F = 1.816, *p* =.169). Although the difference between the pre- and post-tests was significant (F = 43.589, *p* =.000; large effect, η² = 0.331), the control group showed only a small improvement over time.

The group × time interaction showed that the performance changes of both groups were significantly different (F = 43.589, *p* =.000, η² = 0.331, large effect). The experimental group showed a stronger improvement in Left Knee Valgus, whereas the improvement in the control group was more limited.

**Fig. 16:**
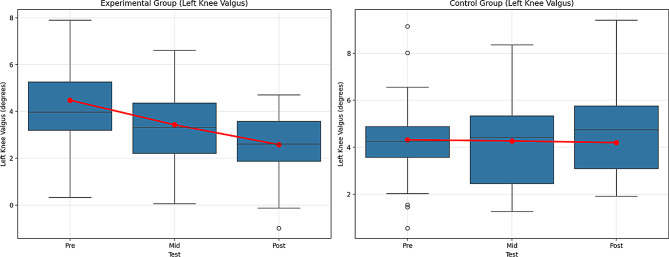
Pre-test, mid-test, and post-test comparisons of Left Knee Valgus performance in (**A**) the experimental group and (**B**) the control group. Red lines represent group mean values

The Bonferroni test revealed significant differences for both groups. For the experimental group, significant differences were found between the pre- and mid-tests (1 > 2), the pre and post-tests (1 > 3), and the mid and post-tests (2 > 3). However, no significant difference was observed between the tests in the control group. Figure 16 shows the graph of the within-group comparison of the pre-test, mid-test, and post-test values of the Left Knee Valgus tests applied to the experimental and control groups.

**Table 15:** Relationships between post-tests in the experimental group

	Tests		CMJ	CMJ Free	DJ	SJ	H.Jump	Right Knee Valgus	Left Knee Valgus
Experimental Group	CMJ	r	1						
		p							
	CMJ Free	r	0.843**	1					
		p	0.000						
	DJ	r	0.798**	0.759**	1				
		p	0.000	0.000					
	SJ	r	0.874**	0.853**	0.778**	1			
		p	0.000	0.000	0.000				
	H.Jump	r	0.467**	0.244**	0.338*	0.271	1		
		p	0.000	0.106	0.023	0.072			
	Right Knee Valgus	r	− 0.391*	− 0.250	− 0.307*	− 0.444*	− 0.449*	1	
		p	0.008	0.097	0.041	0.002	0.002		
	Left Knee Valgus	r	− 0.384*	− 0.162	− 0.220	− 0.222	− 0.424*	0.429*	1
		p	0.009	0.289	0.147	0.144	0.004	0.003	

*p <.05,**p <.001

Table 15 shows the Fisher Z scores for the correlations between the tests performed on the experimental group. The correlation between CMJ and CMJ Free (*r* =.843, *p* <.001) yielded a Z score of 1.2212. The correlation between CMJ and DJ (*r* =.798, *p* <.001) yielded a Z score of 1.0714. The correlation between CMJ and SJ (*r* =.874, *p* <.001) yielded a Z score of 1.3331.

The correlation between CMJ and HJ (*r* =.467, *p* <.001) yielded a Z score of 0.4973. The correlation between CMJ and Right Knee Valgus (*r* = −.391, *p* <.05) yielded a Z score of 0.4118. The correlation between CMJ and Left Knee Valgus (*r* = −.384, *p* <.05) yielded a Z score of 0.4001.

The correlation between CMJ Free and DJ (*r* =.759, *p* <.001) yielded a Z score of 0.9730. The correlation between CMJ Free and SJ (*r* =.853, *p* <.001) yielded a Z score of 1.2562. The correlation between CMJ Free and HJ (*r* =.244, *p* >.05) yielded a Z score of 0.2448. The correlation between CMJ Free and Right Knee Valgus (*r* = −.250, *p* >.05) yielded a Z score of 0.2554. The correlation between CMJ Free and Left Knee Valgus (*r* = −.162, *p* >.05) yielded a Z score of 0.1614.

The correlation between DJ and SJ (*r* =.778, *p* <.001) yielded a Z score of 1.0203. The correlation between DJ and HJ (*r* =.338, *p* <.05) yielded a Z score of 0.3428. The correlation between DJ and Right Knee Valgus (*r* = −.307, *p* <.05) yielded a Z score of 0.3095. The correlation between DJ and Left Knee Valgus (*r* = −.220, *p* >.05) yielded a Z score of 0.2237.

The correlation between SJ and HJ (*r* =.271, *p* >.05) yielded a Z score of 0.2769. The correlation between SJ and Right Knee Valgus (*r* = −.444, *p* <.05) yielded a Z score of 0.4722. The correlation between SJ and Left Knee Valgus (*r* = −.222, *p* >.05) yielded a Z score of 0.2237.

The correlation between HJ and Right Knee Valgus (*r* = −.449, *p* <.05) yielded a Z score of 0.4722. The correlation between HJ and Left Knee Valgus (*r* = −.424, *p* <.05) yielded a Z score of 0.4477.

The correlation between Right Knee Valgus and Left Knee Valgus (*r* =.429, *p* <.05) yielded a Z score of 0.4477.

**Table 16:** Relationships between post-tests in the control group

	Tests		CMJ	CMJ Free	DJ	SJ	H.Jump	Right Knee Valgus	Left Knee Valgus
Control Group	CMJ	r	1						
		p							
	CMJ Free	r	0.814**	1					
		p	0.000						
	DJ	r	0.717**	0.710**	1				
		p	0.000	0.000					
	SJ	r	0.902**	0.844**	0.739**	1			
		p	0.000	0.000	0.000				
	H.Jump	r	0.548**	0.717**	0.406**	0.624**	1		
		p	0.000	0.000	0.006	0.000			
	Right Knee Valgus	r	− 0.418**	− 0.078	− 0.251	− 0.310	− 0.043	1	
		p	0.004	0.609	0.097	0.039	0.778		
	Left Knee Valgus	r	− 0.320*	− 0.096	− 0.256	− 0.218	− 0.011	0.085	1
		p	0.032	0.528	0.090	0.150	0.941	0.579	

*p <.05,**p <.001

Table 16 shows the Fisher Z scores for the correlations between the tests performed on the control group. The correlation between CMJ and CMJ Free (*r* =.814, *p* <.001) yielded a Z score of 1.1270. The correlation between CMJ and DJ (*r* =.717, *p* <.001) yielded a Z score of 0.8872. The correlation between CMJ and SJ (*r* =.902, *p* <.001) yielded a Z score of 1.4722. The correlation between CMJ and HJ (*r* =.548, *p* <.001) yielded a Z score of 0.6042. The correlation between CMJ and Right Knee Valgus (*r* = −.418, *p* <.05) yielded a Z score of 0.4356. The correlation between CMJ and Left Knee Valgus (*r* = −.320, *p* <.05) yielded a Z score of 0.3316.

The correlation between CMJ Free and DJ (*r* =.710, *p* <.001) yielded a Z score of 0.8872. The correlation between CMJ Free and SJ (*r* =.844, *p* <.001) yielded a Z score of 1.2212. The correlation between CMJ Free and HJ (*r* =.717, *p* <.001) yielded a Z score of 0.8872. The correlation between CMJ Free and Right Knee Valgus (*r* = −.078, *p* >.05) yielded a Z score of 0.0701. The correlation between CMJ Free and Left Knee Valgus (*r* = −.096, *p* >.05) yielded a Z score of 0.0902.

The correlation between DJ and SJ (*r* =.739, *p* <.001) yielded a Z score of 0.9287. The correlation between DJ and HJ (*r* =.406, *p* <.05) yielded a Z score of 0.4236. The correlation between DJ and Right Knee Valgus (*r* = −.251, *p* >.05) yielded a Z score of 0.2554. The correlation between DJ and Left Knee Valgus (*r* = −.256, *p* >.05) yielded a Z score of 0.2554.

The correlation between SJ and HJ (*r* =.624, *p* <.001) yielded a Z score of 0.7250. The correlation between SJ and Right Knee Valgus (*r* = −.310, *p* <.05) yielded a Z score of 0.3205. The correlation between SJ and Left Knee Valgus (*r* = −.218, *p* >.05) yielded a Z score of 0.2132.

The correlation between HJ and Right Knee Valgus (*r* = −.043, *p* >.05) yielded a Z score of 0.0400. The correlation between HJ and Left Knee Valgus (*r* = −.011, *p* >.05) yielded a Z score of 0.0100.

The correlation between Right Knee Valgus and Left Knee Valgus (*r* =.085, *p* >.05) yielded a Z score of 0.0000.

## Discussion

The discussion and results of the participants’ jumping performance and knee valgus tests are mentioned in this section. Data on the experimental and control groups’ demographic characteristics (age, height, weight, and BMI) were determined.

When the comparison of the pre-test and post-test data of the experimental and control groups was examined, there was no significant difference between the experimental and control group CMJ test pre-test data. A significant difference was found between the CMJ post-test data of the groups. There was no significant difference between the groups’ CMJ Free pre-test data. There is a significant difference between the CMJ Free post-test data. There is no significant difference between the DJ pre-test data of the groups. There is a significant difference between the DJ post-test data. There is no significant difference between the groups’ SJ pre-test data. There is a significant difference between the SJ post-test data. There is no significant difference between the Horizontal Jump pre-test data of the groups. There is a significant difference between the Horizontal Jump post-test data. The lack of significant differences in the pre-test results suggests that the groups were initially homogeneous. The significant improvements observed in the experimental group in all post-test measurements (CMJ, CMJ Free, DJ, SJ, and Horizontal Jump) support the effectiveness of the applied exercise or training intervention. These results demonstrate that the training program contributed positively to the jumping performance of the football players, while the control group showed no such improvement.

Myer et al. [50] reported in their study that plyometric and balance training applied to high school female athletes reduced the lower extremity valgus angle. In our study, significant decreases in knee valgus angles were observed, particularly in the experimental group. However, while Myer’s study included balance-focused training, our study incorporated strength, flexibility, and plyometric training.

Mandelbaum et al. [12] found a significant reduction in ACL injuries over a two-year period through a program that included strength, balance, agility, plyometric, and proprioceptive exercises. In our study, knee valgus angles decreased in the experimental group, and jumping performance improved. The difference lies in the focus while Mandelbaum’s study targeted long-term injury rates, our study examined short-term performance and biomechanical outcomes.

There is no significant difference between the experimental and control groups in the Right Knee Valgus pre-test data. There is a significant difference between the groups’ Right Knee Valgus post-test data. There is no significant difference between the groups’ Left Knee Valgus pre-test data. There is a significant difference between the groups’ Left Knee Valgus post-test data. Prevention of ACL injuries, which are common in the football branch, which has such an important place in the world, is important for participants to stay in this branch longer and continue their careers healthily. Although there was no difference in the pre-test values ​​of the right and left knee valgus in the experimental and control groups, significant improvement was observed in both knees in the experimental group in the post-tests, indicating that the applied intervention contributed to the prevention of ACL injuries by reducing knee valgus in football players.

Petushek et al. [51] conducted a meta-analysis showing that preventive training programs applied to younger athletes (middle and high school) were more effective in reducing ACL injury risk compared to those implemented in older populations. Similarly, our study demonstrated the positive effects of an intervention in the U19 age group. However, their analysis included only female athletes, whereas our study was conducted with male football players.

The correlation findings, where the relationship between the experimental group tests was analyzed, show that different jumping tests (CMJ, CMJ Free, DJ, SJ, HJ) measure similar physical abilities, and most tests are strongly correlated with one another. In particular, it was observed that the tests requiring explosive strength and jumping capacity reflect each other and evaluate the same physical parameters. However, an inverse relationship was found between the valgus angles of the knee and jumping performance, suggesting that biomechanical deviations in the knee may negatively affect athletic performance. In conclusion, these results indicate that the tests can be used in parallel, but structural issues in the knee should be taken into account during evaluations.

The correlation findings for the control group show that different jumping tests (CMJ, CMJ Free, DJ, SJ, HJ) measure similar physical abilities, with most tests being strongly correlated with one another. Specifically, the tests evaluating explosive strength and jumping capacity were found to reflect similar physical parameters. However, an inverse relationship was observed between the valgus angles of the knee and certain jumping performances, suggesting that knee biomechanical deviations may negatively impact athletic performance. Notably, the correlation between the valgus angles and jumping performance was less pronounced in the control group compared to the experimental group, indicating that the effect of biomechanical issues on performance might vary across groups. Overall, the results suggest that the tests can be used in parallel, but knee structural issues should still be considered during evaluations.

When the CMJ test data of the experimental group were examined, a significant difference was found between the pre-test, the middle test, and the post-test. According to the control group CMJ test data, a significant difference was found between the pre-test, middle, and post-test. It is seen that the increase in the test data of the experimental group is higher than that of the control group. The data show that stretching exercise training, strength exercise training, and plyometric training are effective in increasing participants’ CMJ test values. The most important finding is that the group and time interaction had a significant and large effect. This shows that the improvement in performance of the experimental group was more significant than the control group. As a result, although both groups improved over time, the intervention had a stronger effect on the experimental group. There were significant increases in the CMJ test results of the experimental group between pre-test, interim test and post-test, and the improvement in the experimental group was more pronounced compared to the increase in the control group. This shows that stretching, strength and plyometric training were effective in increasing the vertical jump performance of the participants. Markovic & Mikulic [52] reported that plyometric training can improve CMJ performance by 4–12%. This rate is parallel to the approximately 12.5% improvement observed in the experimental group.

Neilson et al. [25] found that jump landing training supported by feedback effectively reduced both maximum knee flexion angle and ground reaction force. Our study also demonstrated improvements in jump performance and decreases in knee valgus angles. The key difference is that their study incorporated cognitive support (feedback), while our intervention consisted solely of physical exercises.

Cabrejas et al. [53] compared the effects of core and plyometric training with 8 weeks of integrated traditional Rhythmic Gymnasts training (without plyometric training) on opposing motion jump (CMJ) performance; the height of the unilateral and bilateral CMJs increased significantly after integrated core and plyometric training, while no difference in CMJ height was observed after traditional RG training.

When the experimental group’s CMJ Free test data were examined, a significant difference was found between the pre-test, the mid-test, and the post-test. According to the control group CMJ Free test data, a significant difference was found between the pre-test, the mid-test, and the post-test. The group and time interaction was significant, indicating that both groups changed over time but improved at different rates. The results showed that the experimental group improved more rapidly and significantly, while the control group improved less significantly. This corresponds to an improvement of approximately 12.1%, which is quite impressive for short-term interventions according to sports science literature [52–54]. Haynes et al. [40] stated that the intra-class correlation coefficient value obtained through My Jump 2 in the jumping technique dropped by 20 cm to 0.95, decreased by 40 cm, and the value obtained in the jumping technique was 0.98. In their research results [30]. Bogataj et al. [29] found a high intra-class correlation coefficient ranging from 0.92 to 0.96 for 2 in the CMJ jumping technique [39]. Weltin et al. [55] stated that 4 weeks of plyometric training can improve trunk control and reduce abduction moments and knee valgus angle in the training group compared to the control group during the application of lateral cuts, and thus reduce the risk of knee injury. Significant improvements were observed in both the experimental and control groups’ CMJ Free test scores across pre-test, mid-test, and post-test measurements. The reliability of the CMJ measurements using the My Jump 2 application has been supported by previous studies, with high intra-class correlation coefficients reported by Haynes et al. [40] and Bogataj et al. [39]. Additionally, Weltin et al. [55] demonstrated that plyometric training over four weeks enhances trunk control and reduces knee valgus angles, which may contribute to decreased knee injury risk. These findings collectively suggest that the training interventions applied in the experimental group are effective in improving jump performance and potentially lowering injury risk in athletes.

When the DJ test data of the experimental group were examined, a significant difference was found between the pre-test, the mid-test, and the post-test. According to the control group DJ test data, a significant difference was found between the pre-test, the mid-test, and the post-test. When the SJ test data of the experimental group were examined, a significant difference was found between the pre-test, mid-test, and post-test. The group and time interaction was significant, revealing that both groups showed changes, but the experimental group exhibited faster and more pronounced improvement. Faigenbaum et al. [56] reported that 6–8 weeks of plyometric and strength-based training provided significant jumping gains in young individuals due to neuromuscular adaptations, which directly increased functional performance.

According to the control group SJ test data, a significant difference was found between the pre-test, the mid-test, and the post-test. The significant group and time interaction revealed that while both groups changed over time, the experimental group experienced faster and more pronounced improvements. An increased jump height of approximately 14.5% directly affects motor skills, especially explosive power, sprint exit, block start, and short-distance sprint. Markovic & Mikulic [52] reported that plyometric training generally provides 5–10% improvement in SJ and CMJ performance. In this context, an improvement exceeding 14% is highly significant in performance and demonstrates the high effect of the applied intervention. Vaczi et al. [57] in a study conducted with male football players, stated that plyometric training applied to lower extremity strength had positive results [57]. When the horizontal jump test data of the experimental group were examined, a significant difference was found between the pre-test and the mid-test, as well as the pre-test and post-test. Ramírez-Campillo et al. [54] reported that 6–8 weeks of plyometric programs can increase Horizontal Jump distance by 5–10%, and this development is also reflected positively in skills such as sprinting and agility. Filipa et al. [58] reported a significant improvement in composite aspect in young football players in 2012 after an 8-week program for the right and left limbs [58]. Significant improvements were observed in the experimental and control groups across DJ and SJ test results from pre-test to mid-test and post-test measurements. The experimental group showed considerable progress in the horizontal jump test between pre-test and subsequent tests. These findings align with Vaczi et al. [57], who reported positive effects of plyometric training on lower extremity strength in male football players, and Filipa et al. [58], who found significant improvements in young football players’ limb performance following an 8-week training program. The results suggest that the applied training protocols effectively enhance jump performance and lower limb power in football players.

When the experimental group’s Right Knee Valgus test data were examined, a significant difference was found between the pre-test, the mid-test, and the post-test. According to the control group Right Knee Valgus test data, a significant difference was found between the pre-test, the mid-test, and the post-test. The group and time interaction analysis further highlighted that the performance changes between the two groups were significantly different, with the experimental group demonstrating more substantial improvements. In their study, Chappell and Limpisvati [59] examined the kinetic and kinematic fall and jump values before and after training with a three-dimensional motion analysis system. According to the values, there was an increase in knee flexion angles at the moment of fall (*P* =.03), a decrease in dynamic knee valgus moment (*P* =.04), and an improvement in the participants’ right foot and left one-foot jump and vertical jump values (*P* =.01) was observed [59]. It is similar to the results of our literature study. When the experimental group’s Left Knee Valgus test data were examined, a significant difference was found between the pre-test, the mid-test, and the post-test. According to the control group Left Knee Valgus test data, there is no significant difference between the pre-test, the mid-test, and the post-test. The group and time interaction analysis revealed substantial differences in performance changes between the two groups. The experimental group showed a more pronounced improvement, while the control group’s improvements were more modest. Myer et al. [50] emphasized that reductions in knee valgus angle significantly reduce the risk of ACL injury, especially in female athletes, and that strengthening and neuromuscular training programs are effective. Hewett et al. [60] reported that training that improves valgus control increases knee mechanical balance and reduces the risk of injury. Among badminton players, high knee valgus moment and high knee valgus angle have also been associated with higher risks of ACL injuries [61]. The literature supports our study. Herman et al. [28] examined the kinematics of the hips and knees in their research. They found a significant increase in strength in the gluteus medius, quadriceps, gluteus maximus, and hamstring muscles. They stated no considerable difference in the knee and hip kinematics of the control and study groups. He noted that only strength training is insufficient to prevent anterior cruciate ligament injuries, and plyometric, proprioceptive, and balance exercises should be included [28]. Attwood et al. [62] Injury Prevention Exercise Program, consisting of a 42-week program of multiple deterioration and strengthening exercises, balance exercises, landing and cutting exercises (which also required feedback), resulted in a 40% reduction in the incidence of lower extremity injuries compared to the control group. Significant improvements in right and left knee valgus were observed in the experimental group across all test phases, while the control group showed changes only in the right knee. Literature indicates that high knee valgus angles and moments increase ACL injury risk, and that strength training alone is insufficient; plyometric, proprioceptive, and balance exercises should also be included. Long-term, multi-component exercise programs have significantly reduced lower extremity injuries.

The fact that there are different exercise studies in the literature to prevent anterior cruciate injuries supports our study. As in our research, the development and implementation of various preventive programs for the prevention of sports injuries and the examination of the effects of the programs are essential to carry out subsequent training in an up-to-date, more efficient, and scientific manner. Considering the measurement of jumping ability, one of the determinants of athletic performance, and the opposite change in valgus values, both values may be related. Implementing strength, flexibility, and plyometric training programs in addition to regular football training has been shown to affect athlete health positively. These exercise modalities contribute to improved joint stability, particularly in the knee, by reducing lower extremity valgus angles, thereby decreasing the risk of injuries such as anterior cruciate ligament (ACL) ruptures. Moreover, enhanced muscular balance, increased proprioception, and greater flexibility promote better neuromuscular control, which supports injury prevention and overall musculoskeletal health. In this context, the applied training programs improve athletic performance and provide a protective effect against sport-related injuries, supporting long-term athlete well-being. Strength, flexibility, and plyometric training programs enhance athletic performance and improve overall health by supporting posture, movement efficiency, and musculoskeletal function. These programs contribute to better daily functioning, reduced fatigue, improved sleep quality, and overall well-being in participants.

## Conclusion

As a result, at the end of the stretching exercise training, strength training, and plyometric exercise training program applied to the football players in addition to the training, an increase was observed in the CMJ, CMJ Free, DJ, SJ, and horizontal jump values of the football players, while the increase in the experimental group was determined to be higher than the control group. Although it was determined that there was a decrease in right knee valgus values in both the experimental and control groups, the reduction in the experimental group was higher than in the control group. A decrease in left knee valgus values was detected in the experimental group. The reduction in the control group’s left knee valgus values between tests was insignificant. While there was a more significant increase in the jumping forces of the experimental group than the control group, the decrease in valgus values was higher than the control group, which shows that the exercises applied to the participants in addition to the training will increase their jumping performance on the field and at the same time reduce the risk of injury due to valgus.

### Limitations and future research

This study was applied only to football players on the U-19 team. The study can also be conducted on different age groups. The study was conducted only on male football players. It can also be undertaken with female football players by creating a new training program suitable for them. The study was conducted only in Kayseri province. It can also be performed in different provinces, and the results can be compared. In this study, only flexibility, strength, and plyometric training were applied to the experimental group in addition to their regular training. Training programs can be developed and adapted to the specific groups being studied. Researchers can expand the scope of the study by conducting studies with different age groups to diversify preventive training based on the relevant preventive training. Since the variables such as outdoor training, activities, sleep, and nutrition of the participants were uncontrollable factors, they were assumed to be similar and did not affect the test data. In addition, only height, age, body mass index (BMI), and body weight measurements were taken as demographic variables of the participants.

### Suggestions

Coaches can test participants’ knee valgus values at regular intervals to prevent injuries on the field and work to lower valgus values through various strength, flexibility, and plyometric training.

Sports clubs should have their participants’ anthropometric values measured, motor skill tests, and valgus values measured at regular intervals. They should also change their training programs regarding weaknesses due to routine controls.

By conducting regional studies on the characteristics (strength, flexibility) the participants need, coaches can prepare personalized training programs in addition to regular football training, rather than a single, general program.

Participants can be trained about sports injuries and causative factors, and information about training styles and their importance can be provided.

Based on the findings of this study, it is recommended that football players regularly incorporate flexibility, strength, and plyometric exercises into their training programs. These types of exercises are effective in improving jumping performance as well as preventing knee injuries.

Participants who pay attention to knee alignment during technical training and perform regular performance evaluations can make their development process healthier and more controlled. Additionally, structured and consistent training can have positive effects not only physically but also psychologically. In particular, goal setting, positive feedback, and team support can enhance participants’ motivation and strengthen their commitment to the sport.

In future studies, it is important to examine different age groups of young football players based on the findings of this study. Additionally, comparing participants at various performance levels will provide a more comprehensive evaluation of the effectiveness of exercise programs. The effects of participants’ psychological characteristics (such as motivation, stress coping, and self-confidence) on exercise responses should also be considered. Moreover, using different and advanced measurement techniques, such as EMG, three-dimensional motion analysis, and force platforms, will contribute to a more detailed and objective evaluation of program effectiveness. These approaches will expand knowledge in the relevant field and enable the individualization and optimization of training programs.

## Data Availability

The data are available in the Dryad data repository with the URL “http://datadryad.org/share/QZzuZzIghxz333oNhYlN7oj2LulK8ygX1V29o2ChDZU”.
